# Impact of Winter Season on Inpatient Outcomes and Trends in Cardiac Arrest Hospitalizations: A Nationwide Analysis

**DOI:** 10.7759/cureus.79297

**Published:** 2025-02-19

**Authors:** Tochukwu Nzeako, Olawale O Olanisa, Gbolahan Olatunji, Emmanuel Kokori, Nicholas Aderinto, Srihita Patibandla, Adam Barelski, Adedayo A Adeboye

**Affiliations:** 1 Internal Medicine, Christiana Care Health System, Delaware, USA; 2 Internal Medicine, Trinity Health Grand Rapids, Grand Rapids, USA; 3 Public Health, Johns Hopkins Bloomberg School of Public Health, Baltimore, USA; 4 Public Health, University of Ilorin, Ilorin, NGA; 5 Medicine, Ladoke Akintola University of Technology, Ogbomoso, NGA; 6 Cardiovascular Disease, University of Tennessee Health Science Center, Memphis, USA; 7 Cardiovascular Disease, University of Tennessee Health Sciences Center, Memphis, USA

**Keywords:** cardiac arrest, healtchare disparities, inpatient outcome, seasonal variations, winter season

## Abstract

Background: Cardiac arrest presents a critical medical emergency with substantial morbidity and mortality. Seasonal variations, particularly during winter, have been associated with increased cardiovascular risks. However, the impact of winter on inpatient outcomes following cardiac arrest remains underexplored. This nationwide analysis aims to quantify the influence of the winter season on inpatient outcomes and trends in cardiac arrest hospitalizations.

Methods: Data spanning 2016-2020 were extracted from the National Inpatient Sample (NIS) Database Registry. Patients with cardiac arrest were categorized based on hospitalization during winter (November to January) and non-winter (February to October) months. Inclusion criteria, study variables, and outcomes, such as mortality, respiratory failure, interventions, and hospital costs, were assessed. Statistical analyses, including logistic and linear regression models, were employed to determine unadjusted and adjusted outcomes.

Results: Of 1,048,955 cardiac arrest patients, 286,210 were hospitalized during winter. Winter hospitalizations exhibited higher mortality (63.3% vs. 60.9%), even after adjustments (adjusted odds ratio: 1.08; 95% confidence interval 1.05 - 1.11, p<0.001). Reduced odds of advanced interventions (mechanical circulatory support, percutaneous coronary intervention, pacemaker placement) were observed in winter hospitalizations. Unexpectedly, lower hospital costs were associated with winter hospitalizations ($171,115 vs. $177,536, p=0.012). Clinical outcomes (respiratory failure, in-hospital resuscitation, targeted temperature management, hospital length of stay) were comparable between winter and non-winter cohorts. Temporal trends showed an increasing rate of cardiac arrest in both cohorts from 2016 to 2020.

Conclusion: This nationwide analysis reveals the critical impact of winter on inpatient outcomes following cardiac arrest. The findings underscore the urgency of tailored interventions during winter, potential disparities in advanced cardiovascular care, and the need for ongoing research to elucidate economic considerations and optimize patient care strategies.

## Introduction

Cardiac arrest remains a critical medical emergency associated with high morbidity and mortality rates [1]. It accounts for 15%-20% of all natural deaths in adults in the USA and Western Europe and up to 50% of all cardiovascular deaths [1]. While numerous factors influence cardiac arrest outcomes, seasonal variations have garnered increasing attention in recent years [2]. Notably, studies have consistently associated colder temperatures and winter months (WMs) with increased risks of adverse cardiovascular events, including myocardial infarction and cardiac arrest [2,3]. While the association between winter and increased cardiovascular risks is well-documented, the effects of seasonal variations on inpatient outcomes following cardiac arrest remain relatively underexplored [4]. Risk factors for developing cardiovascular diseases such as age, gender, obesity, smoking, hyperlipidemia, hypertension, and diabetes are well established. Yet, when addressing seasonal variations in cardiac arrest outcomes, external factors such as temperature, physical activity, air quality, infections, and diet further define a patient's vulnerability [3,4]. Socio-economically disadvantaged groups, lacking in housing and healthcare, are particularly at risk [3]. Additionally, unsafe working conditions, especially for outdoor workers, increase exposure to extreme temperatures and air pollution [3]. Addressing these variations presents an opportunity to improve patient outcomes.

Exploring the relationship between seasonality and post-cardiac arrest outcomes is crucial in improving cardiovascular health. Understanding how seasonal variations affect the recovery and prognosis of patients who have experienced cardiac arrest can inform physicians in tailoring treatment strategies and resource allocation. In addition, uncovering potential seasonal patterns in post-cardiac arrest outcomes contributes to developing targeted preventive measures and interventions. Moreover, the limited exploration of seasonal influences on inpatient outcomes presents an opportunity for novel research avenues. Investigating whether specific weather conditions or seasonal factors correlate with variations in survival rates, neurological outcomes, or complications during hospitalization could provide valuable insights into optimizing patient care. The primary objective of this nationwide analysis is to explore and quantify the influence of the winter season on inpatient outcomes related to cardiac arrest hospitalizations.

## Materials and methods

Data source and collection 

This study utilized inpatient data from 2016 to 2020 National Inpatient Sample (NIS), a publicly available database developed as part of the Healthcare Cost and Utilization Project (HCUP), which is sponsored by the Agency for Healthcare Research and Quality (AHRQ). As the largest all-payer inpatient database in the USA, the NIS provides comprehensive national and regional estimates of hospital admissions, healthcare utilization, costs, and patient outcomes. The dataset includes detailed discharge records containing patient demographics, primary and secondary diagnoses, procedural information, payer types, and hospital characteristics. Hospitals in the NIS database are categorized based on ownership, teaching status, bed capacity, geographic location, and urban or rural setting. The database uses a 20% probability sample from hospitals across these strata, with discharge records weighted to generate national estimates. Covering data from 48 states participating in HCUP and the District of Columbia, which represents approximately 97% of the US population. Since the dataset is publicly available and de-identified, institutional review board (IRB) approval was not required [5].

Inclusion criteria and study variables

Patients were included in the study if they had a principal diagnosis of cardiac arrest based on the 10th revision of the International Classification of Diseases (ICD)-10 codes. Hospitalizations were grouped according to the month of admission to assess seasonal variations. Patients were further classified into two subgroups: those admitted during the WMs, which included November, December, and January, and those hospitalized during the non-WMs (NWMs) from February through October. Patients under 18 years of age, as well as those for whom the month of admission was missing or unidentifiable, were excluded from the study.

Study population and outcomes

The study population consisted of all eligible hospitalized patients with a primary diagnosis of cardiac arrest recorded in the NIS between 2016 and 2020. Baseline characteristics analyzed included demographics such as age, sex, and race, as well as hospital attributes including bed size, teaching versus non-teaching status, and geographic region, which was categorized as Northeast, Midwest, South, or West. Additionally, comorbid conditions were assessed using the Charlson Comorbidity Index (CCI), a validated tool commonly used in clinical research to predict mortality risk based on pre-existing conditions [5-8]. 

The primary outcome of the study was inpatient mortality. Secondary outcomes included the incidence of respiratory failure, the use of target temperature management (TTM), the need for mechanical circulatory support (MCS) implantation, in-hospital resuscitation rates, pacemaker implantation, mean hospital length of stay (HLOS), and total hospital charges incurred [6]. Furthermore, the study examined trends in cardiac arrest hospitalizations between winter and NWMs over the study period.

Statistical analysis

All statistical analyses were conducted using STATA, version 17.0 NP-Parallel Edition (StataCorp., TX, USA). Continuous variables were compared using independent t-tests, while categorical variables were analyzed using Fisher’s exact test. Logistic regression was employed to evaluate binary and categorical variables, while linear regression was used for continuous variables. A univariate regression model was used to determine unadjusted odds ratios (ORs) for both primary and secondary outcomes, whereas a multivariable logistic and linear regression model was employed to calculate adjusted ORs (aORs) and confidence intervals (CI), accounting for potential confounders. 

Variables with p-values less than 0.1 in univariate analysis were included in the multivariable regression model, with statistical significance determined at a threshold of p < 0.05. The CCI was incorporated into the models to adjust for the comorbidity burden. The NIS database identifies the month of admission using the variable “AMONTH,” where each month is assigned a numerical value ranging from 1 (January) to 12 (December). The final models adjusted for multiple covariates, including age, sex, insurance status, hospital characteristics such as region, bed size, teaching status, nicotine use, comorbidities, baseline oxygen use, and the CCI [5,6,8].

## Results

Baseline characteristics

**Figure 1:**
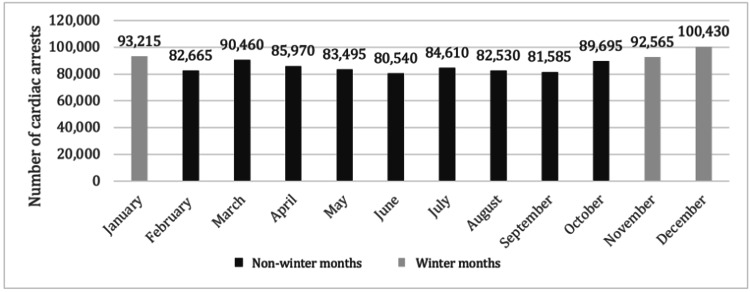
Cumulative number of cardiac arrest hospitalizations per month between 2016 and 2020.

**Figure 2:**
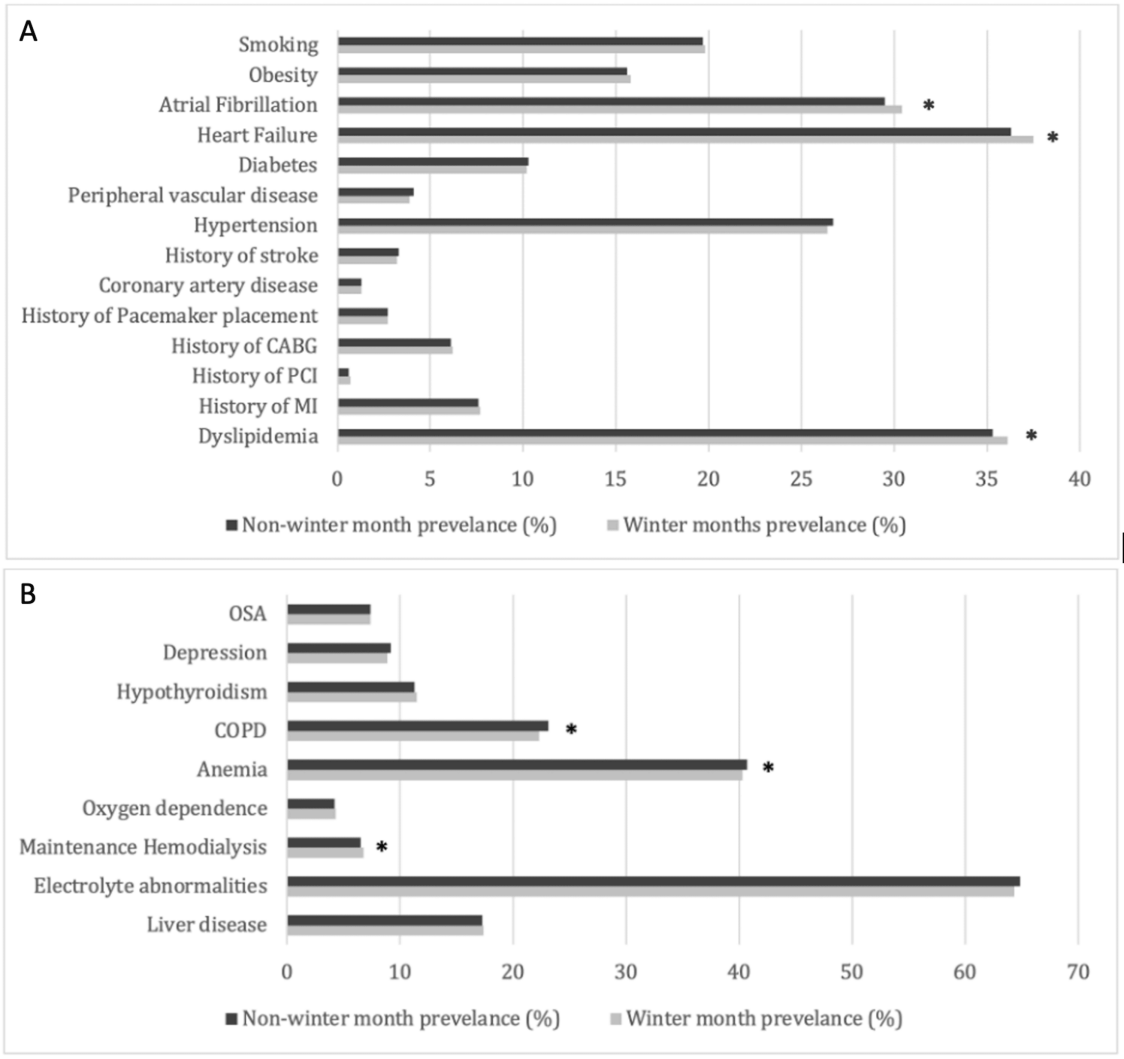
(A) Cardiovascular and (B) non-cardiovascular comorbidities of patients hospitalized with cardiac arrests between 2016 and 2020. * Data comparing winter and non-winter months that has reached statistical significance with p-value < 0.05. MI = myocardial infarction; PCI = percutaneous coronary intervention; CABG = coronary artery bypass graft; COPD = chronic obstructive pulmonary disease; OSA = obstructive sleep apnea

Of the 1,048,955 patients with cardiac arrest analyzed, 286,210 were admitted during WMs. Interestingly, when analyzing the individual months, the WMs had a higher total number of hospitalized patients than the NWMs. Figure 1shows the total number of cardiac arrest hospitalizations that have occurred per month between the years 2016 and 2020. Table 1** **outlines the sociodemographic data of patients included in this study. Patients admitted during WMs were older (65 ± 17.5 years vs. 64 ± 17.9 years) and the majority were males. As seen in Figures 2A, 2B comparing medical comorbidities of hospitalized patients, WM cohorts were more likely to have heart failure (37.5% vs. 36.3, p<0.0001), dyslipidemia (36.1% vs. 35.3%, p=0.0005), atrial fibrillation (30.4% vs. 29.5%) and a history of hemodialysis (6.8% vs. 6.5%, p=0.0266). In contrast, anemia (40.7% vs. 40.3%) and chronic obstructive pulmonary disease (COPD) (23.1% vs. 22.3%) were more prevalent among our NWM cohorts.

**Table 1:** Sociodemographic data for patients hospitalized with cardiac arrests between 2016 and 2020. Data are listed as N (%) unless otherwise stated. Winter months included November, December, and January. Continuous variables were compared using independent t-tests. Categorical variables were analyzed using Fisher’s exact test. Statistical significance was set at a threshold of p < 0.05. MI = myocardial infarction; PCI = percutaneous coronary intervention; CABG = coronary artery bypass graft; COPD = chronic obstructive pulmonary disease; OSA = obstructive sleep apnea

	Cardiac arrests		
Variables	Winter months (N = 286,210)	Non-winter months (N = 761,550)	P-value
Demographic variables			
Male	163,712 (57.2%)	438,653 (57.6%)	0.1211
Female	122,498 (42.8%)	322,897 (42.4%)	0.1211
Age (years), mean ± SE	65 ± 17.5	64 ± 17.9	
Race			0.0368
White	179,740 (62.8%)	477,492 (62.7%)	
Black	56,097 (19.6%)	150,787 (19.8%)	
Hispanic	30,052 (10.5%)	80,724 (10.6%)	
Asian	9,159 (3.2%)	22,085 (2.9%)	
Cardiovascular comorbidities			
Dyslipidemia	103,322 (36.1%)	268,827 (35.3%)	0.0005
History of MI	22,038 (7.7%)	57,878 (7.6%)	0.4939
History of PCI	2,004 (0.7%)	4,569 (0.6%)	0.4549
History of CABG	17,745 (6.2%)	46,455 (6.1%)	0.2302
History of Pacemaker placement	7,728 (2.7%)	20,562 (2.7%)	0.5850
Coronary artery disease	3,721 (1.3%)	9,900 (1.3%)	0.5955
History of stroke	9,159 (3.2%)	25,131 (3.3%)	0.1745
Hypertension	75,559 (26.4%)	203,334 (26.7%)	0.1120
Peripheral vascular disease	11,162 (3.9%)	31,224 (4.1%)	0.1876
Diabetes	29,193 (10.2%)	78,440 (10.3%)	0.6189
Heart Failure	107,329 (37.5%)	276,443 (36.3%)	<0.0001
Atrial Fibrillation	87,008 (30.4%)	224,657 (29.5%)	<0.0001
Obesity	45,221 (15.8%)	118,802 (15.6%)	0.1668
Smoking	56,670 (19.8%)	150,025 (19.7%)	0.7049
Non-cardiovascular comorbidities			
Liver disease	49,800 (17.4%)	131,748 (17.3%)	0.4009
Electrolyte abnormalities	184,033 (64.3%)	493,484 (64.8%)	0.1663
Maintenance Hemodialysis	19,462 (6.8%)	49,501 (6.5%)	0.0266
Oxygen dependence	12,307 (4.3%)	32,747 (4.2%)	0.5116
Anemia	115,343 (40.3%)	309,951 (40.7%)	0.0072
COPD	63,825 (22.3%)	175,918 (23.1%)	0.0005
Hypothyroidism	32,914 (11.5%)	86,055 (11.3%)	0.2097
Depression	25,473 (8.9%)	70,063 (9.2%)	0.0526
OSA	21,180 (7.4%)	56,355 (7.4%)	0.9173
Hospital variables			
Hospital bed-size			0.0301
Small	51,232 (17.9%)	135,556 (17.8%)	
Medium	87,294 (30.5%)	228,465 (30.0%)	
Large	147,398 (51.5%)	397,529 (52.2%)	
Hospital region			0.0002
Northeast	42,932 (15.0%)	117,279 (15.4%)	
Midwest	59,246 (20.7%)	159,164 (20.9%)	
South	125,074 (43.7%)	332,797 (43.9%)	
West	58,959 (20.6%)	150,025 (19.7%)	
Hospital teaching status			0.6339
Non-teaching	77,849 (27.2%)	205,619 (27.0%)	
Teaching	208,361 (72.8%)	555,170 (72.9%)	
Hospital location			0.1634
Rural	17,459 (6.1%)	47,978 (6.3%)	
Urban	268,751 (93.9%)	713,572 (93.7%)	
Charleson Comorbidity Index			<0.0001
1	32,628 (11.4%)	91,386 (12.0%)	
2	47,225 (16.5%)	129,464 (17.0%)	
≥3	206,357 (72.1%)	539,939 (70.9%)	

Unadjusted rates and adjusted odds of in-hospital outcomes 

**Figure 3:**
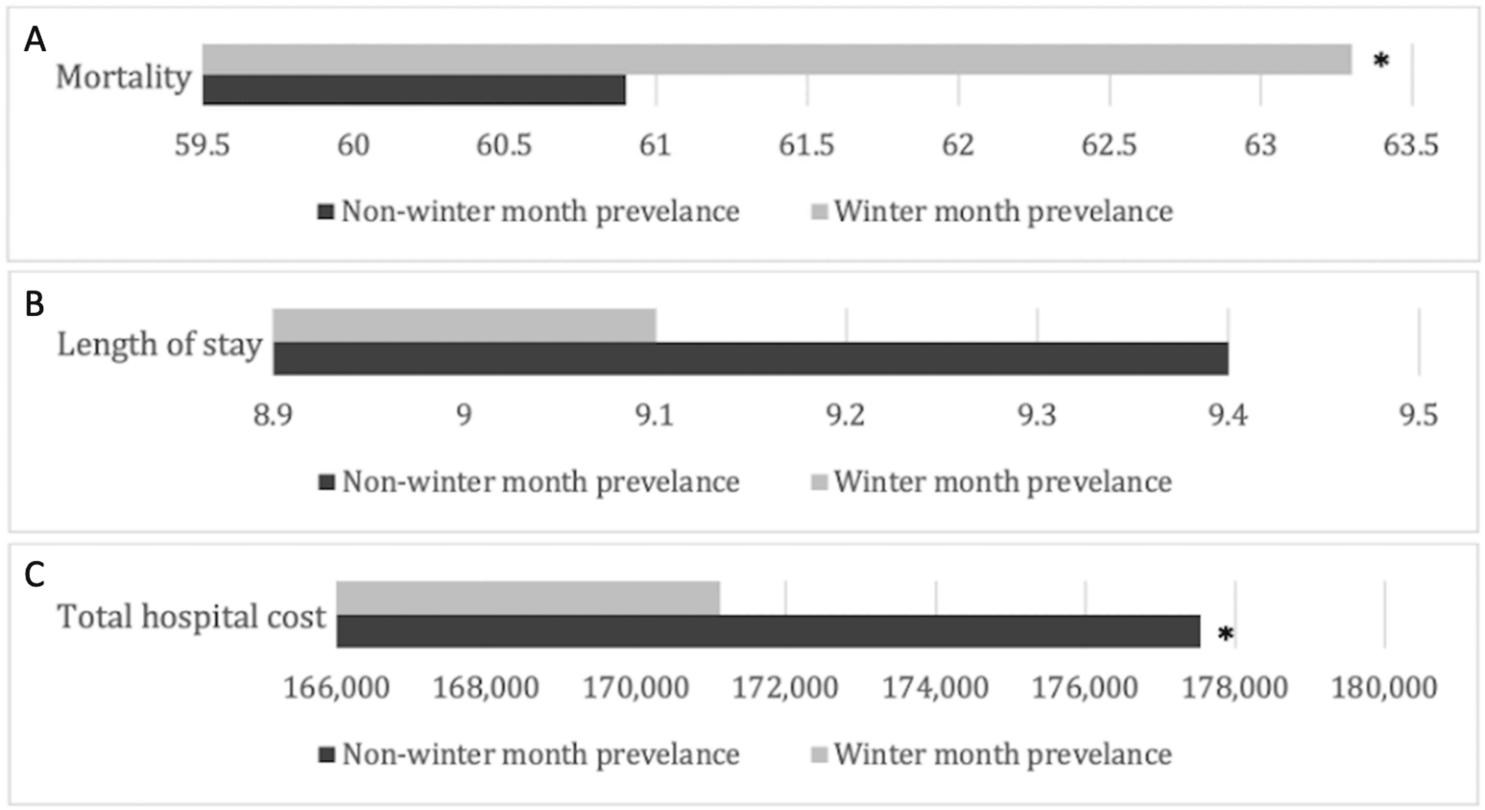
Outcomes following cardiac arrests for patients hospitalized during winter months compared to non-winter months for (A) mortality, (B) length of stay, and (C) total hospital cost. *Data comparing winter and non-winter months that has reached statistical significance with p-value < 0.05.

**Figure 4:**
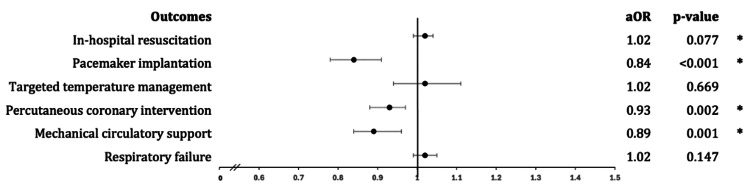
Forrest plot depicting various in-hospital outcomes following cardiac arrest hospitalizations between 2016 and 2020. *Data comparing winter and non-winter months that has reached statistical significance with p-value < 0.05.

In-hospital outcomes of patients hospitalized for cardiac arrests in WMs and NWMs are outlined in Table 2. The mortality rate was higher among hospitalizations during WMs (63.3% vs. 60.9%). This was statistically significant after adjusting for age, sex, race, cardiovascular, non-cardiovascular comorbidities, and hospital characteristics using multivariate analysis (aOR: 1.08; 95% CI 1.05 - 1.11, p<0.001) (Figure 3A). There were no significant differences in the HLOS (9.1 days vs. 9.4 days, p=0.830) (Figure 3B). Our WM hospitalizations were also noted to incur lower hospital costs ($171,115 vs. $177,536, p=0.012) (Figure 3C). Patients hospitalized during WMs were less likely to have MCS implantation (aOR: 0.89; 95% CI 0.84 - 0.96, p=0.001), PCI (aOR: 0.93; 95% CI: 0.88 - 0.97, p=0.002) and pacemaker implantations (aOR: 0.84; 95% CI 0.78 - 0.91, p<0.001) in comparison to NWM hospitalizations with cardiac arrests (Figure 4). The odds of respiratory failure (p=0.147), in-hospital resuscitation (p=0.077), and TTM (p=0.669) were comparable between both cohorts (Figure 4).

**Table 2:** In-hospital outcomes for patients hospitalized with cardiac arrests in winter and non-winter months. Data units are listed as % unless otherwise stated. Winter months included November, December, and January. Continuous variables were compared using independent t-tests. Categorical variables were analyzed using Fisher’s exact test. Statistical significance was set at a threshold of p < 0.05.

Variable	Winter months	Non-winter months	aOR (95% CI)	P-value
Mortality	63.3	60.9	1.08 (1.05 – 1.11)	<0.001
Length of stay (days)	9.1	9.4	0.02 (0.14 – 0.18)	0.830
Total hospital cost ($)	171,115	177,536	3855 (6858 – 852)	0.012
Respiratory failure	65.5	64.9	1.02 (0.99 – 1.05)	0.147
Mechanical circulatory support	3.0	3.2	0.89 (0.84 – 0.96)	0.001
Percutaneous coronary intervention	6.0	6.2	0.93 (0.88 – 0.97)	0.002
Targeted temperature management	1.8	1.7	1.02 (0.94 – 1.11)	0.669
Pacemaker placement	1.9	2.3	0.84 (0.78 – 0.91)	<0.001
In-hospital resuscitation	36.4	35.6	1.02 (0.99 – 1.04)	0.077

Temporal trend

**Figure 5:**
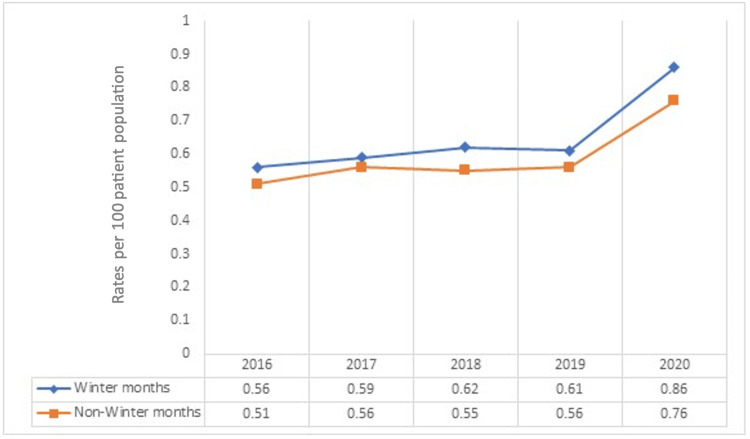
Trends of cardiac arrests per year comparing winter and non-winter months between 2016 and 2020. Data are listed as rates per 100 of the total patient population.

The temporal trends of cardiac arrests throughout the years 2016 to 2020 are illustrated in Figure 5. The rates of cardiac arrest among WM hospitalizations up-trended from 0.56 per 100 patient population in 2016 to 0.62 in 2018, down-trended to 0.61 in 2019, and then rose steeply to 0.86 in 2020. Among NWM hospitalizations, a similar but lower trend was observed and trended from 0.51 per 100-patient population in 2016 to 0.56 in 2017. The rates dropped to 0.55 in 2018, rose to 0.56 in to19, and rose steeply to 0.76 per 100-patient population in 2020.

## Discussion

Our study of inpatient outcomes following cardiac arrests during WM compared to NWM reveals significant implications for clinical practice. A noteworthy observation from our research is the higher total number of hospitalizations during winter compared to NWMs (Figure 1), consistent with previous studies indicating seasonal variations in hospital admissions due to cardiovascular events [4,8]. Examining the demographic characteristics of patients admitted during WM revealed that they were, on average, slightly older than those admitted in NWM (Table 1). This aligns with existing literature highlighting the vulnerability of the elderly population to the physiological stressors associated with colder temperatures [9,10]. Seasonal temperature variations have been linked to increased cardiovascular mortality, particularly among older adults [11,12]. This may be partially attributable to higher baseline prevalence of cardiovascular co-morbidities and disease severity, along with reduced access to healthcare services [12]. Unsurprisingly, a significant rise in cardiac arrests has also been noted in 2020 compared to prior years, presumed to be a result of the COVID-19 pandemic (Figure 5).

The prevalence of males within the winter cohort further indicates the need for targeted preventive measures and interventions, given the potential gender-specific risk factors associated with cardiac emergencies during winter (Table 1). Our findings are similar to those of a study by Kienbacher et al. where low perceived temperature was found to substantially increase the already elevated risk of ST-elevation myocardial infarction (STEMI) in males compared to females [13]. Further, in a study of associations between snowfall duration and risk of MI, the likelihood of MI was increased following snowfall among men but not women [14]. The authors propose that the mechanism responsible for these gender differences may be due to men being more likely to shovel snow and have greater cold exposure compared to women during snowfalls. However, further studies would be needed to evaluate whether differences in hormonal metabolic responses to cold stress among men and women play a role in these findings. Strategies, such as minimizing cold exposure and avoiding sudden physical exertion in cold environments, may help reduce the risk of cardiac arrests and associated complications during winter.

Interestingly, patients admitted during WM exhibited a higher prevalence of heart failure, dyslipidemia, atrial fibrillation, and a history of hemodialysis (Figures 2A, 2B). Recent studies have explored the relationship between temperature changes and lipid levels, revealing significant associations that may influence cardiovascular risk. A study by Halonen et al. found that with each 5°C increase in ambient temperature, there was a decrease in high-density lipoprotein (HDL) levels and an increase in low-density lipoprotein (LDL) levels [15]. Jin et al. also found a nearly linear association between temperature variations and the risk of dyslipidemia in middle-aged and elderly people [16]. These changes in lipid profiles are important because they can affect the risk of coronary heart disease and other cardiovascular events. Although these studies show that higher temperatures were more likely to be associated with dyslipidemia, our research revealed the alternative, warranting further investigation. For atrial fibrillation, cold-induced fluctuations of sympathetic and parasympathetic tones, heart rate variability, and increased blood pressure, may increase cardiac workload and result in increased arrhythmic burden during WMs [17]. Little research has been done investigating the effects of extreme temperatures on chronic kidney disease or heart failure. Overall, these physiologic changes illustrate the connection between cardiovascular health, winter-related stressors, and pre-existing conditions. Preventive strategies, such as enhanced monitoring of heart failure patients, optimizing lipid management, and addressing atrial fibrillation risks, should be explored to reduce adverse cardiac outcomes during colder months.

The observed higher mortality rate among hospitalizations during WMs shows the critical nature of cardiac emergencies during colder seasons (Figures 3A-3C). Importantly, this association remained statistically significant even after adjustments for potential confounders. Our observation of a higher mortality rate during winter hospitalizations is consistent with the findings of studies that have reported increased cardiovascular events and mortality during colder seasons [10,18,19]. This aligns with the broader understanding that adverse weather conditions, physiological stressors, and potential exacerbation of pre-existing conditions contribute to heightened cardiovascular risks during winter [20]. In a systematic review and meta-analysis by Fan et al., every decrease in temperature of 1°C increased cardiovascular disease-related mortality by 1.6% [21]. Mortality related to cardiovascular disease was found to have the most profound association with cold temperatures. Cold temperatures may influence cardiovascular mechanics through increased blood viscosity, platelet aggregation, metabolic derangements, insulin resistance, and changes in red blood cell count, all of which increase the risk of ischemic heart disease and stroke [21]. The authors propose that the association between cold temperatures and cardiac arrest morbidity may also be explained by cold-induced autonomic nervous system dysfunction and coagulation cascade activation [21]. Furthermore, seasonal variations in physical activity, dietary habits, and respiratory infections (such as influenza) can further amplify cardiovascular risk during WMs [22,23]. Season-specific preventive strategies may be beneficial in reducing cold-related cardiovascular risks and mortality.

In addition, patients hospitalized during WMs were less likely to receive crucial cardiac interventions than their NWM counterparts. The reduced odds of MCS implantation, PCI, and pacemaker implantations during WMs suggest potential disparities in the delivery of advanced cardiovascular care (Figure 4). Another plausible consideration is that in winter, the overall number of patients experiencing cardiac arrest due to non-cardiovascular diseases increases, leading to a reduced need for interventions targeted toward treating cardiovascular diseases. Identifying reduced odds for advanced cardiovascular interventions during winter hospitalizations contributes novel insights to existing literature. While previous studies have hinted at delays in cardiac procedures during winter, our findings suggest potential disparities in the delivery of advanced cardiovascular care [24,25]. Understanding the factors contributing to these disparities is crucial for developing targeted interventions to ensure equitable access to life-saving treatments throughout the year. An unexpected finding was the lower hospital costs associated with WM hospitalizations. This finding introduces a novel dimension to the discussion and deviates from the limited information in previous literature. While some studies have explored the economic impact of seasonal variations in healthcare, the specific observation of lower costs during winter hospitalizations requires further investigation [24,25]. These findings may partially be attributed to factors such as survival rates during WMs and length of stay.

In contrast to the variations observed in mortality rates and treatment modalities, our study reveals comparable odds of respiratory failure, in-hospital resuscitation, TTM, and HLOS between winter and non-winter hospitalizations (Figures 3A-3C, 4). This aligns with certain aspects of the existing literature, where some studies have reported stable clinical outcomes across seasons, irrespective of variations in mortality or treatment patterns [3,10]. Despite differences in mortality and interventions, the stability observed in clinical outcomes aligns with previous studies emphasizing the importance of consistent, high-quality care irrespective of seasonal variations.

Limitations and strengths of the study

This study relies on data obtained from the NIS Database Registry, which has inherent limitations. The accuracy and completeness of the data are contingent on the reporting practices of individual hospitals, potentially leading to underreporting or misclassification of variables. In addition, the study's retrospective design limits the establishment of causal relationships. While associations between the winter season and outcomes are identified, causation cannot be definitively inferred, and the influence of unmeasured confounders remains a possibility. However, using the NIS Database provides a large, nationally representative sample, enhancing the external validity of the findings and allowing for insights into broader trends in cardiac arrest hospitalizations. The study categorizes hospitalizations based on WMs and NWMs but does not incorporate actual temperature or weather conditions (e.g., extreme cold spells, snowfall, or humidity). This limits the ability to directly correlate specific environmental stressors with outcomes. Further, the COVID-19 pandemic introduced significant confounders during the year 2020, as the peak of the pandemic occurred during the WMs, and many cardiac arrests that occurred were likely associated with these infections. The study evaluates inpatient outcomes, including mortality, interventions, economic considerations, and clinical outcomes. This comprehensive approach provides a nuanced understanding of the multifaceted impact of the winter season on cardiac arrest hospitalizations.

## Conclusions

This study highlights increased cardiac arrest mortality in winter, likely due to disparities in advanced interventions such as MCS, PCI, and pacemaker implantation. The unexpected finding of lower hospital costs during winter warrants further investigation into resource allocation and healthcare utilization patterns. Despite seasonal differences in mortality and intervention rates, clinical outcomes such as respiratory failure, in-hospital resuscitation, TTM, and HLOS remained stable. This consistency underscores the need for year-round optimization of cardiac care to ensure equitable access and improve patient outcomes regardless of season.
